# Two Archaeal RecJ Nucleases from *Methanocaldococcus jannaschii* Show Reverse Hydrolysis Polarity: Implication to Their Unique Function in Archaea

**DOI:** 10.3390/genes8090211

**Published:** 2017-08-24

**Authors:** Gang-Shun Yi, Yang Song, Wei-Wei Wang, Jia-Nan Chen, Wei Deng, Weiguo Cao, Feng-Ping Wang, Xiang Xiao, Xi-Peng Liu

**Affiliations:** 1 State Key Laboratory of Microbial Metabolism, School of Life Sciences and Biotechnology, Shanghai Jiao Tong University, 800 Dong-Chuan Road, Shanghai 200240, China; 13166228531@163.com (G.-S.Y.); www1037554814@sjtu.edu.cn (W.-W.W.); 14330101150369@sjtu.edu.cn (J.-N.C.); fengpingw@sjtu.edu.cn (F.-P.W.); xoxiang@sjtu.edu.cn (X.X.); 2 National Center for Protein Science, Chinese Academy of Sciences, Shanghai 201204, China; songyang@sibcb.ac.cn (Y.S.); dengwei@sibcb.ac.cn (W.D.); 3 Department of Genetics and Biochemistry, Clemson University, Clemson, SC 29634, USA; wgc@clemson.edu; 4 State Key Laboratory of Ocean Engineering, School of Naval Architecture, Ocean and Civil Engineering, Shanghai Jiao Tong University, 800 Dong-Chuan Road, Shanghai 200240, China

**Keywords:** archaeal RecJ, Cdc45-MCM-GINS, nuclease, GINS, interaction

## Abstract

Bacterial nuclease RecJ, which exists in almost all bacterial species, specifically degrades single-stranded (ss) DNA in the 5′ to 3′ direction. Some archaeal phyla, except Crenarchaea, also encode RecJ homologs. Compared with bacterial RecJ, archaeal RecJ exhibits a largely different amino acid sequence and domain organization. Archaeal RecJs from *Thermococcus kodakarensis* and *Pyrococcus furiosus* show 5′→3′ exonuclease activity on ssDNA. Interestingly, more than one RecJ exists in some Euryarchaeota classes, such as Methanomicrobia, Methanococci, Methanomicrobia, Methanobacteria, and Archaeoglobi. Here we report the biochemical characterization of two RecJs from *Methanocaldococcus jannaschii*, the long RecJ1 (MJ0977) and short RecJ2 (MJ0831) to understand their enzymatic properties. RecJ1 is a 5′→3′ exonuclease with a preference to ssDNA; however, RecJ2 is a 3′→5′ exonuclease with a preference to ssRNA. The 5′ terminal phosphate promotes RecJ1 activity, but the 3′ terminal phosphate inhibits RecJ2 nuclease. Go-Ichi-Ni-San (GINS) complex does not interact with two RecJs and does not promote their nuclease activities. Finally, we discuss the diversity, function, and molecular evolution of RecJ in archaeal taxonomy. Our analyses provide insight into the function and evolution of conserved archaeal RecJ/eukaryotic Cdc45 protein.

## 1. Introduction

Nucleases, including endonuclease and exonuclease, play important roles in DNA recombination and repair, degradation and recycling of DNA and RNA, and maturation of RNA and Okazaki fragments [1]. RecJ is a kind of nuclease involved in three DNA repair pathways: homologous recombination, mismatch repair (MMR), and base excision repair. RecJ nuclease belongs to the DHH phosphodiesterase superfamily with a conserved signature motif DHH. DHH motif is consisted of three successive conserved residues located at the corresponding N-terminal DHH domain, and the DHHA motif, located at the corresponding C-terminal domain, is a typical signature motif for classifying family of the DHH phosphodiesterase superfamily. Based on the sequence difference of the DHHA motif, the DHH phosphodiesterase superfamily can be split into DHHA1 and DHHA2 groups. The DHHA1 group has a typical DHHA1 motif of GGGHXXAAG, whereas the DHHA2 group lacks this typical motif or has a divergent or atypical motif. Based on the difference in biochemical properties and conserved motifs, DHH phosphodiesterases are classified into four families. Family 1 includes prokaryotic RecJ nuclease and eukaryotic Cdc45 protein [1,2,3,4], Family 2 is composed of various nanoRNases (Nrn), including NrnA [5] and NrnB [6], which specifically degrade short single-stranded (ss) RNA molecule [5,6,7]. Family 3 degrades the nucleotide derivatives, but not oligonucleotides, and includes eukaryotic Prune and PPX1 [8] and prokaryotic family II inorganic pyrophosphatase [9]. Family 4, HAN nuclease [10], is a fused protein containing an N-terminal domain and the C-terminal DHH phosphodiesterase domain, and is specific to archaea kingdom.

The family 1 DHH phosphodiesterase includes three subfamilies: bacterial RecJ, archaeal RecJ and eukaryotic Cdc45 protein. RecJ has a typical DHHA1 motif, but Cdc45 does not. Bacterial RecJ nuclease shows both single-stranded DNA (ssDNA)-specific 5′→3′ exonuclease and deoxyribose phosphatase (dRPase) activities [3]. Its ssDNA-specific 5′→3′ exonuclease is responsible for generating a long 3′ ssDNA for strand invasion in homologous recombination [11], or a long ssDNA gap for DNA resynthesis by DNA polymerase in MMR [12]. The 5′ dRPase of RecJ removes deoxyribose phosphate of the single-strand break generated by the cleavage of an abasic site by apurinic/apyrimidinic (AP) endonucleases in base excision repair [13]. Structurally, most bacterial RecJs, such as *Escherichia coli* RecJ, feature an N-terminal catalytic core, which consists of two domains of DHH and DHHA interconnected by a long helix, and a C-terminal oligonucleotide/oligosaccharide-binding (OB) domain that improves ssDNA-binding capability. Some bacterial RecJs, such as the RecJs of *Thermus thermophilus* and *Deinococcus radiodurans*, have an additional C-terminal domain [14,15]. The C-terminal domain IV of *D. radiodurans* RecJ (DrRecJ) can increase the 5′→3′ nuclease activity by promoting ssDNA substrate binding and interacting with the HerA helicase [15].

Compared with the bacterial RecJ nuclease, little is known about archaeal RecJ nucleases. Research on archaeal RecJs mainly focused on their 5′→3′ exonuclease activity on ssDNA [2,16], 3′→5′ exonuclease activity on single-stranded RNA (ssRNA), and mismatched ribonucleotide of RNA/DNA hybrids [17]. The 3′→5′ exonuclease on RNA possibly removes 3′-mismatched ribonucleotides from the RNA primers in chromosomal DNA replication or is involved in the degradation of diverse ssRNAs [17]. The two potential *recj* genes from *Methanocaldococcus jannaschii* DSM 2661 can supply the capability of DNA recombination repair in an *recj*-deleted *E. coli* strain [16]. Unlike bacterial RecJs, archaeal RecJ nucleases only have two domains corresponding to the bacterial catalytic core domains of DHH and DHHA but lack the OB domain [14,17,18]. Moreover, archaeal RecJ proteins are longer by approximately 100 amino acid residues than the bacterial RecJ catalytic core domain [17]. This additional sequence forms a single domain, the minichromosome maintenance (MCM)-binding domain (MBD), in the topological structure of archaeal RecJ from *Thermococcus kodakarensis* [19], and occupies a location similar to the OB-fold domain of DrRecJ and *T. thermophilus* RecJ (TthRecJ) [14,20].

Despite the broad distribution of RecJ nuclease in bacteria and archaea, RecJ homolog does not exist in eukaryotes. Cdc45, an essential replication initiation protein whose site-mutations result in partial defect in DNA replication [21], shows low-sequence similarity to the conserved catalytic core of the RecJ nuclease subfamily; however, Cdc45 lacks most of the conserved motifs and residues that are essential for prokaryotic enzymatic activity [4,22]. Despite the lack of nuclease activity, Cdc45 retains ssDNA- and ssRNA-binding capability and functions as molecular wedge for DNA unwinding [22,23]. Recently, three groups of researchers reported their results on the structures of bacterial RecJs, archaeal RecJs, and human Cdc45 protein [19,20,24]. These proteins exhibited a similar overall topology, indicating their evolution from a common ancestor.

In addition to nuclease activity, archaeal RecJ also interacts with some subunits of DNA replisome, such as the Go-Ichi-Ni-San (GINS) complex, a central component in the archaeal DNA replication fork and replicative MCM helicase [2,25,26], to form a multi-subunit complex RecJ-MCM-GINS (RMG) [19,25]. Similar to archaeal RecJ, eukaryotic Cdc45 also interacts with MCM2–7 and GINS to form a complex Cdc45-MCM-GINS (CMG), which is believed to act as a DNA helicase during chromosome replication [27,28]. The crystal structure of human Cdc45 and cryo-electron microscopy (EM) structure of CMG provide not only a better understanding of the mechanism of subunit interaction in the CMG complex [24,29,30], but also clues regarding the subunit interaction in RMG [19].

BLAST with the *Pyrococcus furiosus* RecJ as a query sequence, it identified more than one RecJ gene in some archaea genomes, especially the methane-producing species. Previous works also found out the diversity of archaeal RecJ [16,31]. During the preparation of our manuscript, Ishino and coworker reported the biochemical characterization of two RecJs from *Thermoplasma acidophilum* [31]. TacRecJ1 is a ssDNA specific 5′exonuclease, and TacRecJ2 is a 3′ exonuclease on both ssDNA and ssRNA. On the two RecJ nucleases from *M. jannaschii*, although they were primarily characterized [16], the protein preparations were largely impure, just the cell extract of an *E. coli* that was deleted the *recj* gene and supplied with one of two *M. jannaschii recj* genes [16]. To fully understand the enzymatic properties of two *M. jannaschii* RecJs, we recombinantly expressed, purified and biochemically characterized them in detail. Both RecJs are single-stranded DNA/RNA specific nucleases. RecJ1 (MJ0977) is a 5′→3′ exonuclease with a weak preference to DNA, and RecJ2 (MJ0831) is a 3′→5′ exonuclease with a preference to RNA. The terminal phosphate affected enzymatic activity differently. The 5′ terminal phosphate promotes RecJ1 activity, but the 3′ terminal phosphate inhibits RecJ2 nuclease. The GINS does not interact with either RecJ and thus does not promote their nuclease activities on ssDNA and ssRNA. Finally, the diversity, function in DNA repair, and molecular evolution of RecJ in archaeal taxonomy are discussed. Our results provide new clues to understand the functions of archaeal RecJ in nucleic acid metabolism and its evolution relationship with bacterial RecJ and eukaryotic Cdc45 protein.

## 2. Materials and Methods

### 2.1. Materials

KOD-plus DNA polymerase was purchased from Toyobo (Osaka, Japan). Nickel–nitrilotriacetic acid resin was purchased from Bio-Rad (Hercules, CA, USA). RNase A inhibitor was purchased from Takara (Shiga, Japan). Oligodeoxyribonucleotides and oligoribonucleotides (Table S1) were synthesized by Invitrogen (Carlsbad, CA, USA) and Takara (Shiga, Japan), respectively. The expression vectors of pDEST17 (Invitrogen) and pET28-sumo were used throughout this study. *E. coli* strain DH5α was used in the gene cloning and Rosetta 2(DE3)pLysS (Novagen) strain was used to express recombinant protein. All other chemicals and reagents were of analytic grade.

### 2.2. Preparation of Recombinant Proteins

Genes encoding for the archaeal RecJ nucleases (MJ0831 and MJ0977) and GINS (MJ0248) were amplified from *M. jannaschii* genomic DNA by polymerase chain reaction (PCR) using their respective primers (Table S1) and then inserted into pDEST17 or pET28-sumo, as described previously [17]. Amino acid substitutions were introduced into RecJs by PCR-mediated mutagenesis using KOD-plus DNA polymerase and the appropriate primers (Table S1). Nucleotide sequences were confirmed by DNA sequencing.

Recombinant plasmids were introduced into the Rosetta 2(DE3)pLysS strain of *E. coli* to express recombinant proteins. The expressions of recombinant proteins were induced by 0.5 mM isopropylthio-β-galactoside. The recombinant proteins were purified via immobilized Ni^2+^ affinity chromatography. The affinity purification was performed as follows: bacterial pellet was suspended in lysis buffer (20 mM Tris-HCl, pH 8.0; 0.3 M NaCl, 5 mM mercaptoethanol, 5 mM imidazole, 1 mM phenylmethylsulfonyl fluoride, and 10% glycerol) and then disrupted by sonication. After incubation for 30 min at 65 °C (not conducted for MjaGINS-sumo protein), cell extract was clarified by centrifugation at 12,000× *g* for 30 min. After loading the supernatant onto a column pre-equilibrated with lysis buffer, the resin was washed with >25 column volumes of lysis buffer containing 20 mM imidazole. Finally, bound proteins were eluted from the column using elution buffer (20 mM Tris-HCl, pH 8.0; 0.3 M NaCl, 5 mM mercaptoethanol, 200 mM imidazole, and 10% glycerol). After verifying the purity of eluate using 15% sodium dodecyl sulfate polyacrylamide gel electrophoresis (SDS-PAGE), samples were dialyzed against a storage buffer (20 mM Tris-HCl, pH 8.0; 0.1 M NaCl, and 50% glycerol) and stored in small aliquots at −20 °C.

### 2.3. Characterization of Methanocaldococcus jannaschii Enzymes

MJ0831 and MJ0977 were characterized in a standard reaction buffer consisting of 20 mM Tris-HCl (pH 7.5), 50 mM NaCl, 1 mM dithiothreitol (DTT), 2.0 mM MnCl_2_, and 100 ng/μL BSA before optimization. Then, the pH value, ions strength, reaction temperature, and divalent ions were optimized on the basis of standard reaction buffer. Table S1 presents the oligoribonucleotides and oligodeoxyribonucleotides used in exonuclease activity assays. The dependence of activity on substrate structures on was characterized using ssDNA, double-stranded DNA (dsDNA), and dsDNA with 3′ or 5′ overhang. The effect of MjaGINS on two MjaRecJ nucleases was determined by assaying nuclease activity in the presence of increasing concentrations of MjaGINS. After incubation for a specified time at 50 °C, an equal volume of stopping buffer (90% formamide, 100 mM EDTA, and 0.2% SDS) was added to the reaction. Subsequently, the reactions were subjected to 15% 8 M urea-denatured polyacrylamide gel electrophoresis (PAGE). After electrophoresis, images of the gels were quantitated using FL5000 Fluorescent Scanner (FUJIFILM, Tokyo, Japan).

### 2.4. Determining the Interaction between MjaRecJs and MjaGINS

Two experiments were used to identify the possible interactions of the two MjaRecJs and MjaGINS. First, co-purification of RecJ and GINS was conducted to determine the interaction between RecJs and GINS. During co-purification, the purified MjaRecJ with a 6×His tag was mixed with the GINS, whose 6×His tag and sumo domain were removed by Uip protease, in a molecular ratio of 1:2. The mixtures were incubated at 37 °C for 30 min to form the possible complex. If GINS interacts with RecJ, It will be co-purified by the 6×His-RecJ. Second, RecJ and GINS were purified separately and then mixed in a molecular ratio of 1:2 to permit the formation of a possible complex. To check the existence of RecJ–GINS complex, Gel filtration chromatography was performed using a Hiload Superdex 200 column (GE Healthcare, Pittsburgh, PA, USA) pre-equilibrated with 20 mM HEPES (pH 7.0), 100 mM NaCl, 1 mM DTT, 0.1 mM EDTA, and 2% glycerol.

## 3. Results

### 3.1. Substrate Preferences of two MjaRecJs

**Figure 1:**
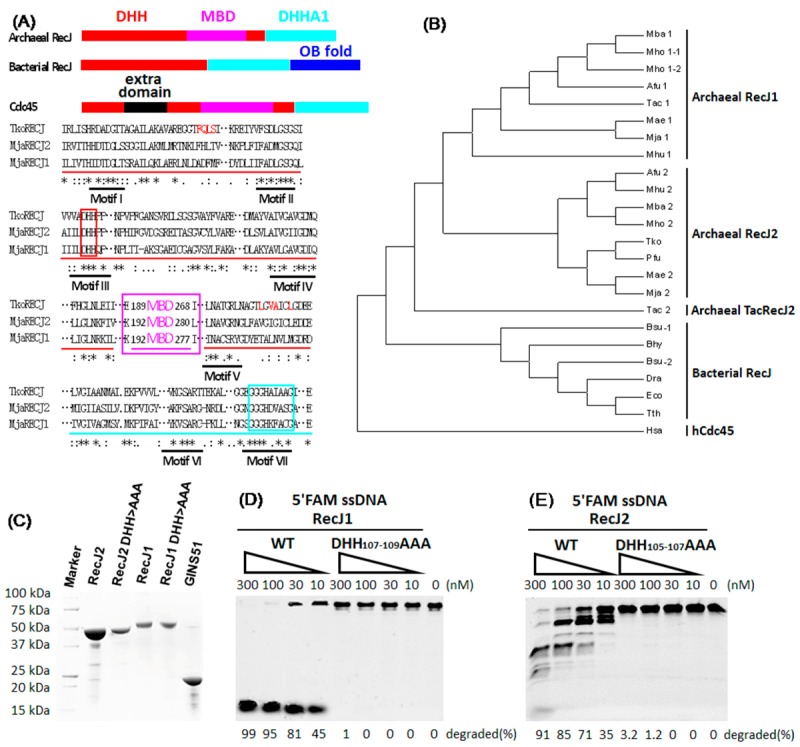
Two RecJs from *Methanocaldococcus jannaschii* demonstrate nuclease activity. (**A**) Multi-alignment of three archaeal RecJs. The domain combinations of archaeal and bacterial RecJs, and eukaryotic Cdc45 protein is compared on the top of panel A, and the names of each domain are indicated. For multi-alignment of RecJs, red, purple and cyan lines are used to represent the domains of DHH, MBD, DHHA, respectively. The conserved motifs are marked by black lines, and the motifs of DHH and DHHA1 are highlighted with red and cyan box, respectively. The middle domain, the MCM helicase Binding Domain (MBD) and its boundary are highlighted by a purple box with the indicated number of residues. The residues responsible for interaction with GINS51 subunit are shown in red. (**B**) The phylogenetic tree of RecJ homologs is built based on multi-alignment of these sequences. Archaeal RecJ1 and RecJ2 are classified based on their sequence similarity to *M. jannaschii* RecJ1 and RecJ2. RecJ homologs come from archaea of *Methanocaldococcus jannaschii* DSM 2661 (Mja), *Methanococcus aeolicus* Nankai-3 (Mae), *Methanospirillum hungatei* JF-1 (Mhu), *Methanosarcina barkeri* strain (str.) Fusaro (Mba), *Thermoplasma acidophilum* DSM 1728 (Tac), *Archaeoglobus fulgidus* DSM 4304 (Afu), *Methanomethylovorans hollandica* DSM 15,978 (Mho), *Pyrococcus furiosus* DSM 3638 (Pfu), and *Thermococcus kodakarensis* KOD1 (Tko); bacteria of *Deinococcus radiodurans* R1 (Dra), *Thermus thermophilus* HB8 (Tth), *Bacillus subtilis* str. 168 (Bsu), *Escherichia coli* K12 (Eco), and *Brachyspira hyodysenteriae* WA1 (Bhy); human Cdc45 (Hsa). (**C**) Expression and affinity purification of five *M. jannaschii* recombinant proteins. Increased amounts of wild-type (WT) or DHH motif mutated MjaRecJ1 (**D**) or MjaRecJ2 (**E**) were incubated with 200 nM 23 nt 5′FAM-labeled ssDNA substrates at 55 °C for 20 min in a standard reaction buffer. The degraded amount of substrate was quantified and listed at the bottom of the panel.

Some archaeal species, such as *M. jannaschii* DSM 2661, contain more than one RecJ gene. Both RecJs of *M. jannaschii* have classical domain combinations, including the MBD domain specific to archaeal RecJ and Cdc45 protein. Two *M. jannaschii* RecJs, MjaRecJ1 (MJ0977), and MjaRecJ2 (MJ0831), have lower sequence similarity of approximately 30%, and show lower similarity to *T. kodakaraensis* RecJ (TkoRecJ), which is the only RecJ nuclease in *T. kodakaraensis* (Figure 1A). The two MjaRecJs have seven conserved motifs (I–VII), such as DHH (motif III) and DHHA1 (motif VII), which are common among many DHH phosphodiesterase families. TkoRecJ and MjaRecJs are different with regard to the conserved residues responsible for interacting with GINS (Figure 1A, red). A complete phylogenetic analysis of RecJs showed that RecJ1 and RecJ2 from some archaeal groups belong to two different branches. The RecJs from some archaea that contain a single *recj* gene such as TkoRecJ and *P. furiosus* RecJ (PfuRecJ), belong to the RecJ2 subfamily (Figure 1B). The bacterial RecJs form a distinct evolutionary branch that does not belong to any of archaeal RecJ groups.

To understand their enzymatic function, the two MjaRecJ proteins were recombinantly expressed, purified and biochemically characterized (Figure 1C). Activity assays confirmed that both MjaRecJs showed nuclease activity on ssDNA in opposite direction. *M. jannaschii* RecJ1 was probably a 5′→3′ exonuclease (Figure 1D), and MjaRecJ2 was probably a 3′→5′ exonuclease (Figure 1E). Their hydrolysis polarity was further confirmed using phosphothioate-modified substrates in next section. Changing the conserved motif the DHH to three alanines deprived the nuclease activity, indicating that DHH motif is essential for the nuclease activity (Figure 1D–E).

**Figure 2:**
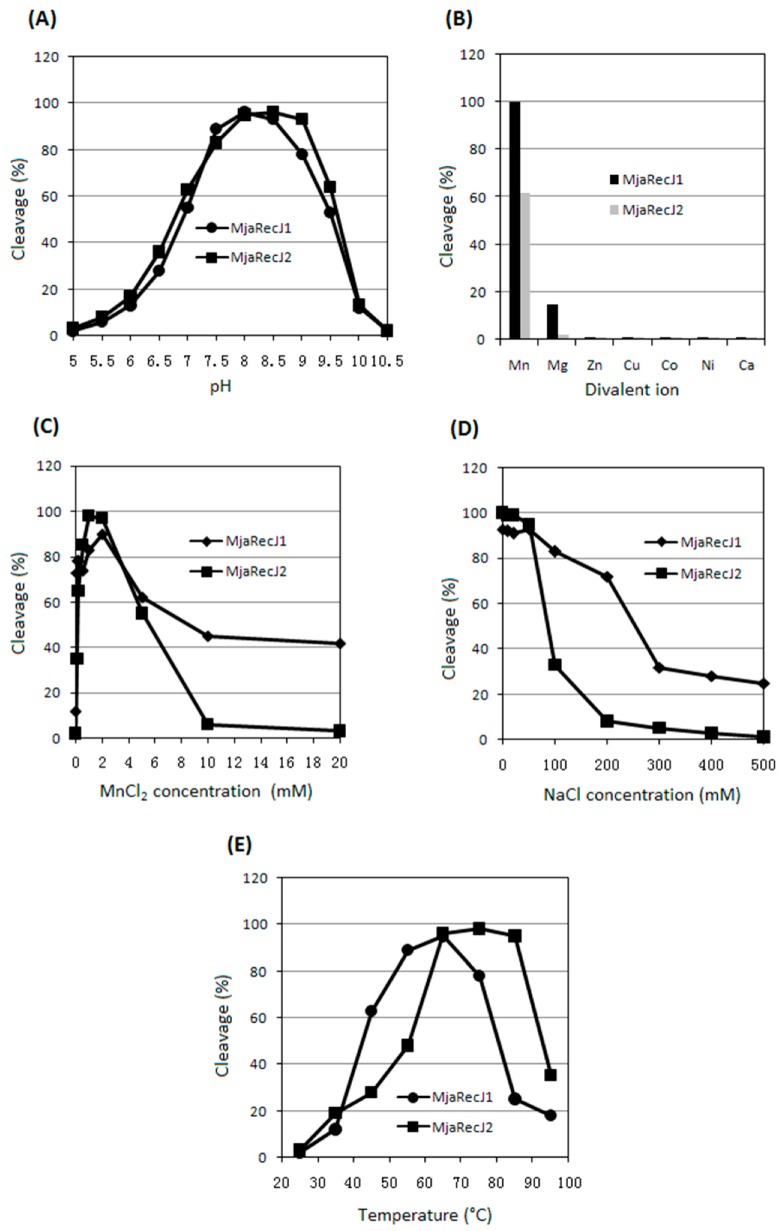
Optimization of ssDNA hydrolysis by MjaRecJs. pH value (**A**), divalent ions (**B**), concentration of divalent manganese ions (**C**), ion strength (**D**), and reaction temperature (**E**) were optimized for nuclease activities of two MjaRecJs (40 nM MjaRecJ1 or 50 nM MjaRecJ2) using a 23 nt single-stranded DNA (ssDNA) as substrate (200 nM). The degraded amount of substrate DNA was quantified and plotted vs. each value.

After positive detection of the nuclease activity, which is consistent with previous results [16], optimal reaction parameters with regard to pH, ion strength, divalent ions, and reaction temperature were determined for the two MjaRecJs (Figure 2). The RecJs displayed the highest activity at pH 8.0 (MjaRecJ1, Figure S1A) and 8.5 (MjaRecJ2, Figure S2A). Divalent ion manganese Mn^2+^ was the most effective metal cofactor (Figure S1B or Figure S2B), with the optimal concentration at 2.0 mM for MjaRecJ1 (Figure S1C) and 1.0 mM for MjaRecJ2 (Figure S2C), respectively. The two RecJs showed higher activity at lower concentrations of NaCl (Figure S1D or Figure S2D). Their optimal reaction temperatures differed. MjaRecJ1 and MjaRecJ2 showed the highest activities at 65 °C (Figure S1E) and 85 °C (Figure S2E), respectively. MjaRecJ2 is more thermostable than MjaRecJ1 (Figure S3); the result is consistent with those for the optimal reaction temperatures.

**Figure 3:**
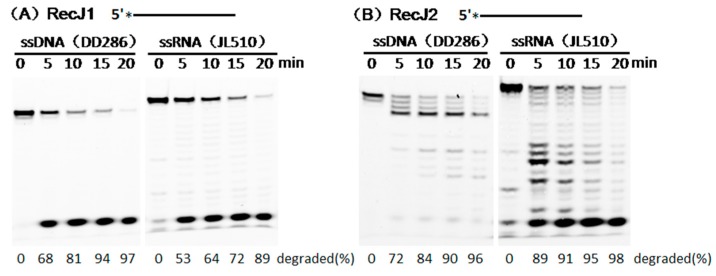
Preferences for (deoxy)ribose of two MjaRecJs. Reactions were performed at 55 °C with increasing time using 200 nM 23 nt 5′-FAM labeled ssDNA and ssRNA as substrates in their respective reaction buffer. 40 nM MjaRecJ1 and 50 nM MjaRecJ2 were used, respectively, in each reaction. The degraded amount of substrate was quantified at each time and listed at the bottom of the panel.

Since some nucleases hydrolyze both DNA and RNA, the (deoxy)ribose dependency of the two RecJs were characterized using ssDNA and ssRNA as substrates. MjaRecJs had a different (deoxy)ribose dependence as compared with bacterial RecJ, which only hydrolyzes ssDNA [3,14,15,20]. MjaRecJ1 could hydrolyze both ssDNA and ssRNA from the 5′ side (Figure 3A). MjaRecJ2 favored ssRNA hydrolysis with a clearly increased rate as compared with ssDNA substrate (Figure 3B). Therefore, the different (deoxy)ribose preferences of two MjaRecJs may suggest their different roles in nucleic acid metabolism in vivo.

### 3.2. Hydrolysis Polarity of Two MjaRecJs

**Figure 4:**
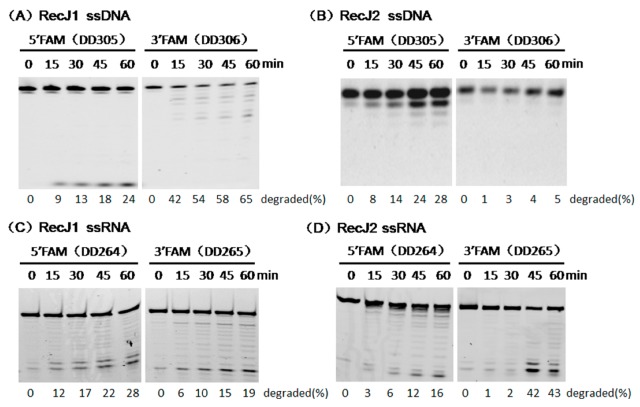
Hydrolysis polarities of two MjaRecJ nucleases. Two MjaRecJs (1.5 μM MjaRecJ1 or 10 μM MjaRecJ2) were incubated with fully phosphothioate-modified 200 nM 23 nt ssDNA or 17 nt ssRNA substrates in their respective reaction buffer at 55 °C with increasing time. SsDNA and ssRNA are labeled with fluorescence group fluorescein FAM at 5′ or 3′ end, respectively. The degraded amount of substrate was quantified at each time and listed at the bottom of the panel.

The fully phosphothioate-modified ssDNA and ssRNA were used as substrates to verify in detail the hydrolysis direction of two MjaRecJs. The existence of phosphothioate groups largely decreased the enzymatic hydrolysis rate and allowed capturing the image of each product during substrate degradation. For ssDNA degradation by MjaRecJ1 (Figure 4A), DNA ladders were generated from the 3′-FAM-labeled ssDNA, and only 1 nt products were generated from the 5′-FAM-labeled ssDNA. These results demonstrated that MjaRecJ1 degraded ssDNA from the 5′ end, and 5′-FAM group did not inhibit the hydrolysis of the first 5′ phosphodiester bond. For ssDNA degradation by MjaRecJ2 (Figure 4B), DNA ladders were generated from 5′-FAM-labeled ssDNA, and products did not appear for 3′-FAM-labeled ssDNA. These results confirmed that MjaRecJ2 degraded ssDNA in the 3′→5′ direction, and the 3′-FAM group strongly inhibited the hydrolysis of the 3′ first phosphodiester bonds. The degradation of fully-phosphothioate-modified ssDNA also showed that RecJ1 was more processive than RecJ2 (Figure 4A,B). For the fully phosphothioate-modified ssRNAs, MjaRecJ1 degraded ssRNA in the 5′→3′ direction (Figure 4C), and MjaRecJ2 degraded ssRNA in the 3′→5′ direction (Figure 4D). In summary, MjaRecJ1 was a 5′ exonuclease on both ssDNA and ssRNA, and MjaRecJ2 was a 3′ exonuclease on both ssDNA and ssRNA.

Using the partially phosphothioate-modified ssDNA as substrate, the two MjaRecJs also showed the same manner of degradation (Figure S4). The phosphothioate groups at the 5′ end clearly blocked the hydrolysis of ssDNA by MjaRecJ1 (Figure S4A; lanes 6, 10 and 12). When several phosphothioate groups exist at the 3′ end, they strongly blocked the degradation of ssDNA by MjaRecJ2 (Figure S4B; lanes 4, 6 and 12). The internal successive phosphothioate groups strongly hindered degradation before the modifications by MjaRecJs (Figure S4, lanes 4 and 10).

### 3.3. Opposite Effect of Terminal Phosphate Groups on MjaRecJs Activity

**Figure 5:**
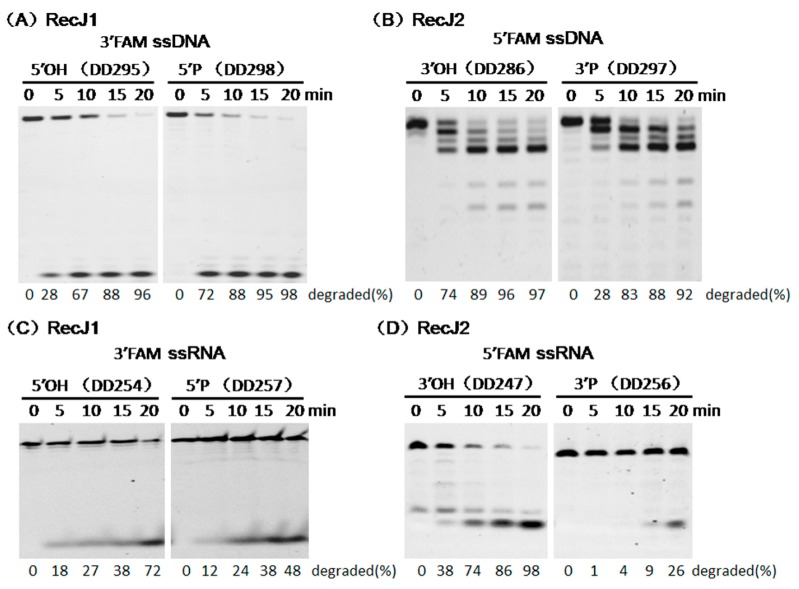
Effect of terminal phosphate group on *M. jannaschii* RecJ activity. Two MjaRecJs (40 nM RecJ1 or 50 nM RecJ2) were incubated with 200 nM 23 nt ssDNA or 12/16 nt ssRNA substrates in their respective reaction buffer at 55 °C with increasing time. The substrates are labeled with fluorescence group FAM at 5′ or 3′ end, and have 3′ or 5′ terminal phosphate groups. The degraded amount of substrate was quantified at each time and listed at the bottom of the panel.

The terminal phosphate group generally affected exonuclease activity [32]. We characterized the effect of phosphate groups on the exonuclease activity of the two MjaRecJs (Figure 5). The 5′ phosphate group clearly promoted MjaRecJ1 activity on ssDNA (Figure 5A) but weakly affected the ssRNA substrate (Figure 5C). In contrast to MjaRecJ1, MjaRecJ2 showed a largely decreased activity on 3′-phosphorylated ssDNA and ssRNA (Figure 5B or Figure 5D). Furthermore, the 3′-phosphorylated ssRNA substrate displayed a more intensive inhibition than ssDNA.

Interestingly, 3′-phosphorylated ssDNA and ssRNA inhibited the 5′ exonuclease activity of MjaRecJ1 (Figure S5A, lane 4). Although MjaRecJ2 did not show clear 5′ exonuclease activity on ssDNA and ssRNA with a 5′-OH terminus (Figure S5B, lane 6), distinct 5′ exonuclease activity was observed on 5′-phosphorylated ssDNA and ssRNA (Figure S5B, lane 8). These results suggested that MjaRecJ2 may also function as a 5′ exonuclease especially on 5′-phosphorylated DNA.

### 3.4. Preferred Substrate Length of MjaRecJs

**Figure 6:**
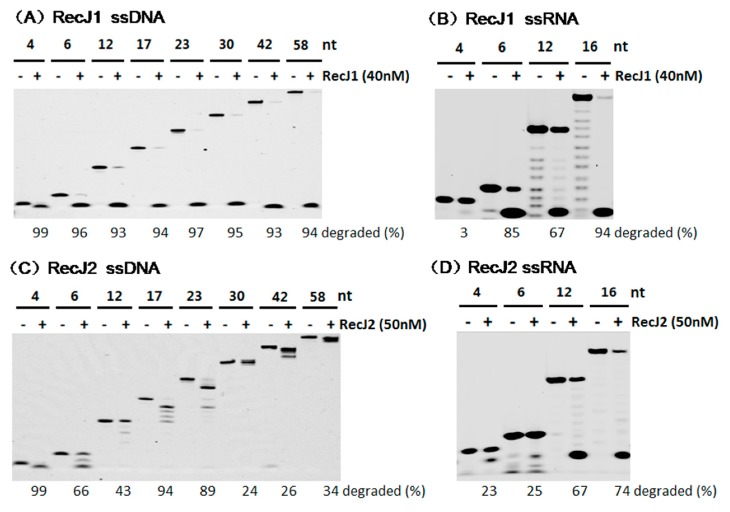
Substrate length preferences of MjaRecJs. Two MjaRecJs (40 nM MjaRecJ1 or 50 nM MjaRecJ2) were incubated with 200 nM 5′-FAM-labeled ssDNA or ssRNA with different lengths as substrates in their respective reaction buffer at 55 °C for 20 min. The degraded amount of substrate was quantified at each time and listed at the bottom of the panel.

The two MjaRecJs had different preferences for substrate length. MjaRecJ1 could hydrolyze all length ssDNA (Figure 6A), and ssRNA ≥ 6nt (Figure 6B). MjaRecJ2 was preferable to shorter ssDNAs, which were degraded with extremely low efficiency when longer than 23 nt (Figure 6C). Only ssRNAs that are 12 or 16nt could be hydrolyzed, with a higher efficiency than ssDNA, by MjaRecJ2, and shorter ssRNAs (4 and 6 nt) are degraded from the 3′ end with very lower efficiency.

### 3.5. Strand Preferences of MjaRecJs

**Figure 7:**
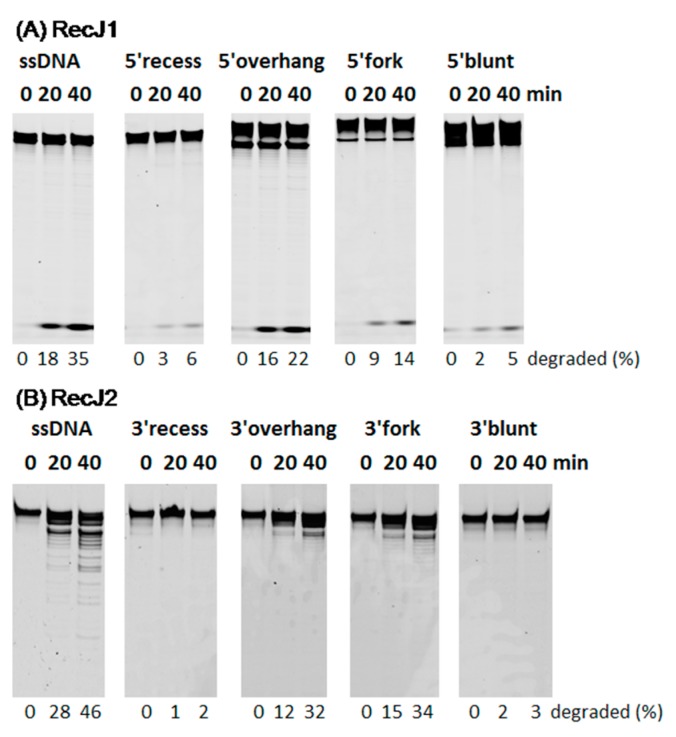
Selectivity of two MjaRecJs on DNA secondary structure. Two MjaRecJs (40 nM MjaRecJ1 or 50 nM MjaRecJ2) were incubated with 200 nM DNA substrates at 55 °C for 0, 20, and 40 min in their respective reaction buffer. DNA secondary structures are single-stranded, forked, overhanged, recessed, and blunt. The degraded amount of substrate DNA was quantified at each time and listed at the bottom of the panel.

Provided that the two MjaRecJs could efficiently hydrolyze ssDNA, double-stranded (ds) DNAs with different single-stranded structures were used to observe MjaRecJs activity on these molecules. Our results showed that ssDNA was the most favored substrate of two MjaRecJs (Figure 7). MjaRecJ1 hydrolyzed DNAs in the order of ssDNA > 5′ overhang > 5′ fork > 5′ blunt ≈ 5′ recess, and MjaRecJ2 followed the order of ssDNA > 3′ fork ≈ 3′ overhang >> 5′ blunt ≈ 5′ recess.

### 3.6. No Interaction between MjaRecJs and MjaGINS

**Figure 8:**
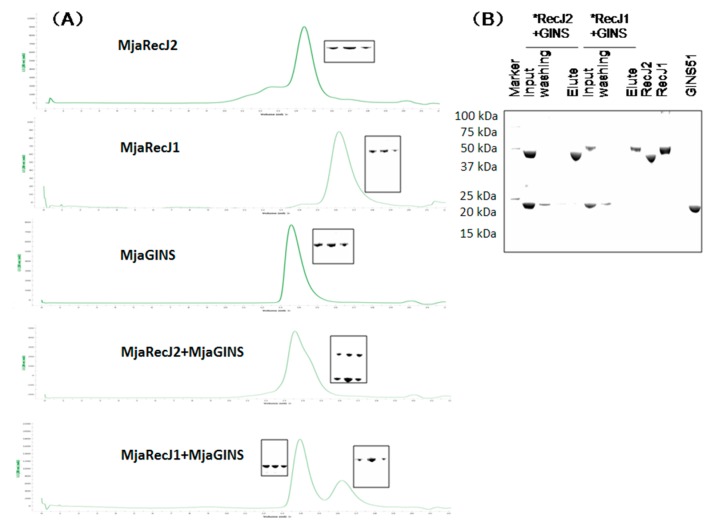
*Methanocaldococcus jannaschii* GINS does not interact with any of MjaRecJs. (**A**) Gel filtration was used to characterize the existence of complexes between MjaGINS51 and MjaRecJ1 or MjaRecJ2. Each peak was collected for protein identification by sodium dodecyl sulfate polyacrylamide gel electropheresis (SDS-PAGE). (**B**) The pulldown experiments were used to characterize the interaction between *M. jannaschii* GINS and RecJ1 or RecJ2. 6×Histine-Tag RecJs were co-purified with the no His-Tag GINS using a Ni-NTA Resin. His-Tag MjaRecJ1 or MjaRecJ2 was mixed with MjaGINS (His-Tag free) in a molecular ratio of 1:2. After binding and washing, and resins were eluted with buffer containing 200 mM imidazole. Protein(s) in elutes were verified by 15% SDS-PAGE.

Considering that archaeal RecJ nuclease, TkoRecJ, forms a complex with the GINS [2,19,26], we characterized the possible interaction between MjaRecJs and MjaGINS. Surprisingly, both MjaRecJs did not form a complex with MjaGINS. However, the work on the RecJs from *Thermoplasma acidophilum* showed that TacRecJ2 forms a complex with GINS, but TacRecJ1 not [31]. Our result on MjaRecJ1 is consistent to that of TacRecJ1, but MjaRecJ2 showed the result contrary to TacRecJ2. The retention time of the mixtures of MjaRecJ1 and GINS were similar to that of any of the two proteins alone, indicating a lack of interaction between MjaRecJ1 and MjaGINS (Figure 8A). The mixtures of MjaRecJ2 and GINS did not generate a peak that moved faster than that of any of alone protein (Figure 8A), indicating that there was no interaction between MjaRecJ2 and MjaGINS. Another possibility is that MjaRecJ2 and GINS do interact but the complex takes a changed conformation and with the similar elution time to those of MjaRecJ2 or GINS. Surprisingly, MjaRecJ2 might exist in dimer based on its elution time (Figure 8A). It is possible that the dimer of MjaRecJ2 hinders its interaction with MjaGINS, for example, the dimer interface occupies the interaction surface for interacting with MjaGINS. Pulldown experiments using the mixtures of MjaRecJ and MjaGINS also confirmed that both MjaRecJs did not form a complex with MjaGINS (Figure 8B). However, the PfuRecJ forms a stable complex with PfuGINS (Figure S6A). Pulldown experiments using the induced *E. coli* cells co-expressing the MjaRecJ and MjaGINS further confirmed that no clear interaction existed between MjaRecJ and MjaGINS (Figure S6A). Since the tagged proteins were used in the pulldown experiment, the tag might have a potential negative effect on protein-protein interactions. Meanwhile, we also did a Microscale Thermophoresis (MST) experiment, an analysis technology for protein interaction. Our MST experiments also confirmed that the interaction does not exist between GINS and MajRecJs (Figure S6B). By checking the residues of MjaRecJs and TkoRecJ, we observed that the residues interacting with GINS have changed largely in MjaRecJ1, but they retained the most conservation in MjaRecJ2. *T. kodakaraensis* GINS51 promotes RecJ nuclease activity via forming a complex [2,19]. Consistent to the interaction results, MjaGINS51 had no promotion on the activities of two MjaRecJs (Figure S7), indirectly supporting the result that the two MjaRecJs do not interact with MjaGINS.

## 4. Discussion

### 4.1. Important Evolutionary Marker of Archaeal RecJ and Cdc45

Compared with bacterial RecJ, archaeal RecJ and hCdc45 possess a separate domain, namely the MBD domain (Figure 1A), that locates between the motifs IV and V of the DHH domain and participates in the interaction with MCM binding [13,19]. Although human Cdc45 and TkoGAN exhibit the similar structural fold [13,19], when compared with archaeal RecJ, hCdc45 has an additional sequence inserted between motifs III and IIIa of the DHH domain [17,19]. Perhaps, both mutations of the conserved residues and insertion of two additional domains caused Cdc45 to evolve into a protein that specifically binds ssDNA and prevents occasional slippage of the leading strand from the core channel of the CMG complex [33], or interacts with other DNA replication proteins, such as Sld3 [34]. It is also possible that a nuclease activity is at the heart of the ancestral replisome [35].

The similarity of crystal structures and conserved motifs may aid in the elucidation of the evolutionary origins of the RecJ/Cdc45 subfamily. It can be speculated that the ancestor of the RecJ/Cdc45 protein might originally evolve into bacterial and archaeal RecJ branches. Then, the bacterial RecJs had evolved into specific nucleases by adding the OB-fold domain. The archaeal RecJ branch, except for functioning as a nuclease in archaea, also had evolved into Cdc45 by inserting another domain between motifs III and IIIa. For archaea with two RecJs, the ancestor archaeal RecJ split into two groups: 3′ and 5′ exonucleases. The RecJ phylogenetic tree showed that TkoRecJ and PfuRecJ belongs to the archaeal RecJ2, but not RecJ1 the subfamily (Figure 1B). However, TkoRecJ and PfuRecJ have a nuclease activity similar to MjaRecJ1, but not to MjaRecJ2. Since TacRecJ2 interact with GINS, it is possible that the GINS-interaction characteristic makes PfuRecJ and TkoRecJ, which both interact with GINS, are more similar to archaeal RecJ2.

### 4.2. Hydrolysis Polarity of Archeal RecJs

The diversified hydrolysis polarity of archaeal RecJs might be universal in archaea. More than one archaea species possesses two or more *recJ* genes. We selected three other species, *Thermoplasma acidophilum*, *Archeoglobus fulgidus*, *and Methanococcus voltae*, to characterize the enzymatic activities of their RecJ homologs. For *T. acidophilum* we confirmed the 5′ exonuclease of RecJ1 and the 3′ exonuclease of RecJ2 [31]. The two RecJ1s from *A. fulgidus* and *M. voltae* were also 5′ exonuclease specific on ssDNA, while we could not identify any nuclease activity of their RecJ2s.

Both TacRecJ1 and TkoRecJ demonstrated 5′→3′ exonuclease activity only on ssDNA [2,31]. PfuRecJ, a homologue with higher sequence similarity to TkoGAN, can also hydrolyze ssDNA and ssRNA in the 5′→3′ and 3′→5′ direction [17], respectively. However, MjaRecJ1 hydrolyze both ssDNA and ssRNA in the 5′→3′ direction. To understand the hydrolysis polarity and its evolution in the DHH phosphodiesterase superfamily, the co-crystal structure of archaeal RecJ and ssDNA or ssRNA should be determined to characterize their catalytic mechanism.

### 4.3. Function of MjaRecJs in Archaeal DNA Replication and Repair

Both euryarchaeal and crenarchaeal GINS form a stable complex with archaeal Cdc45 homologs (RecJ in the former and RecJdbh in the latter) [2,19,25,36]. However, we did not confirm the interaction between MjaGINS and its two RecJs. Since the two MjaRecJs did not form a complex with MjaGINS, it suggests that the MjaRecJs do not participate in unwinding the chromosomal DNA during DNA replication. In future more experiments should be conducted to confirm whether an in vitro or in vivo interaction exists between MjaRecJs and GINS. On the other hand, the knockout of two *recj* genes in archaea, which has genetic operation tools, should be done to confirm their functions in vivo based on the corresponding phenotypes of mutants.

Similar to the Eukaryotic CMG complex, archaea also have a complex RecJ-MCM-GINS (RMG) [25]. Updates to the function of RMG are still unknown. In Crenarchaea, RMG possibly functions as replicative DNA helicase [36]. The six-subunit complex of heterogenous GINS tetra-subunits and RecJdbh (namely, GC complex in a ratio of 2:2:2) in Crenarchaea *Sulfolobus* is specifically located in the replicative fork, indicating that the complex is essential for DNA replication [36]. However, the euryarchaeal *Haloferax volcanii* did not require the *recj* gene for its normal growth [37]. Recent works on *T. kodakarensis* also demonstrated that GAN could be deleted with no discernable effects on viability and growth, indicating that it is not essential to the archaeal MCM replicative helicase [38].

Since MjaRecJ1 and MjaRecJ2 can complement the function of the deleted *recj* gene during DNA recombination repair in *E. coli* [16], they also probably function in DNA repair processes, such as recombination repair, similar to that in bacterial RecJ [39]. Considering the existence of several different DNA resection pathways in prokaryotes [40,41], the two RecJs possibly undergo two-directional resection during the recombination repair of dsDNA break in *M. jannaschii*. TkoGAN might participate in primer removal during Okazaki fragment maturation cooperated with Fen1 and RNase HII. Failing in deleting both Fen1 and GAN genes suggested that both enzymes catalyze primer removal in vivo as a nuclease [38]. Similar to GAN, MjaRecJ1 might remove the RNA primer by its 5′-exonuclease on the flapped RNA section of Okazaki fragment. Since MjaRecJ2 has more pronounced 3′ exonuclease activity on ssRNA than on ssDNA; thus, it also may be responsible for degrading diverse abnormal ssRNAs (such as fragmental RNAs), as observed for the nanoRNase of DHH phosphodiesterase superfamily [5,6,7]. Therefore, more studies should be conducted to confirm the importance of *recj* and *gins* genes in archaeal DNA replication and repair and to determine the functional diversity of archaeal RecJ and GINS homologs, especially in archaea with two RecJs and only one GINS51 subunit.

In summary, on the basis of identification of nuclease activity by Rajman & Lovett [16], we have further confirmed the reverse hydrolysis polarity of two MjaRecJs that are ideal models for investigating the molecular mechanism to determine the hydrolysis direction using structural and biochemical approaches. Meanwhile, the two MjaRecJs are also good models for studying the evolutionary pathway of archaeal RecJ and eukaryotic Cdc45 protein, and for elucidating the functions of RecJs in DNA replication and repair.

## Supplementary Materials

The following are available online at www.mdpi.com/2073-4425/8/9/211/s1. Figure S1 Biochemical characterization of MjaRecJ1. Figure S2 Biochemical characterization of MjaRecJ2. Figure S3 Thermostabilities of MjaRecJs. Figure S4 Hydrolysis polarity of two MjaRecJs confirmed by special phosphothioate-modified substrates. Figure S5 Effect of terminal phosphate group on *M. jannaschii* RecJs activity. Figure S6 Interaction identification of MjaRecJs and MjaGINS. Figure S7 Effect of MjaGINS on MjaRecJs activity. Table S1 Oligonucleotides used in this research.
