# Supplementary material for: Two Archaeal RecJ Nucleases from Methanocaldococcus jannaschii Show Reverse Hydrolysis Polarity: Implication to Their Unique Function in Archaea
Source: Genes (Basel). 2017 Aug 24;8(9):211. doi: 10.3390/genes8090211 (PMC5615345; doi:10.3390/genes8090211)
Supplement: Supplementary file 1 [file genes-08-00211-s001.docx]

**Supplementary Data**

**Supplementary Figures 1-6**


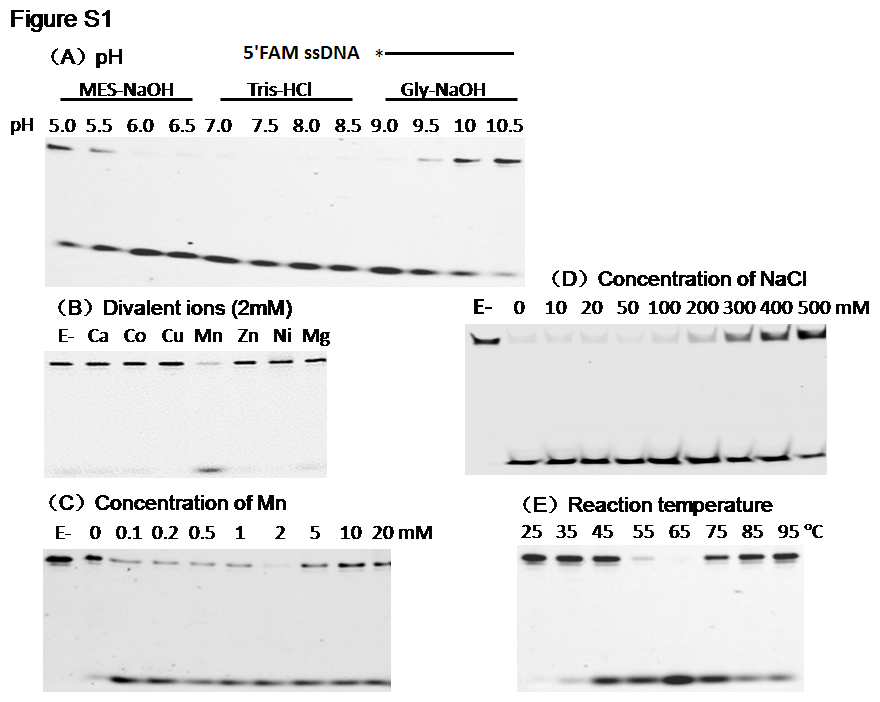


**Figure S1. Biochemical characterization of MjaRecJ1.** Reaction parameters, including pH (A), divalent ions (B), concentration of Mn^2+^ (C), ion strength (D), and reaction temperature (E), were optimized using a 23 nt ssDNA as substrate. During biochemical characterization of 5’ 🡪3’ exonuclease activity, the ssDNA substrate (100 nM) was incubated with 40 nM MjaRecJ1 at 55 °C (except for the assay of reaction temperature) for 20 min. The pH of reaction buffer was optimized in basic buffer consisting of 50 mM NaCl, 1 mM DTT, and 2.0 mM MnCl_2_. After optimization of pH, effects of divalent ions (2.0 mM) on activity were characterized in buffer with pH 8.5, and optimal concentration of Mn^2+^ was further assayed within the range of 0.1–20 mM. Ion strength was optimized in the buffer consisting of 20 mM Tris-HCl (pH 8.0), 2.0 mM Mn^2+^, 1 mM DTT, and increasing concentrations of NaCl. Finally, reaction temperature was characterized in an optimized buffer consisting of 20 mM Tris-HCl (pH 8.0), 50 mM NaCl, 2.0 mM Mn^2+^, and 1 mM DTT. Products in each image were quantified to construct the curve shown in Figure 2.

**
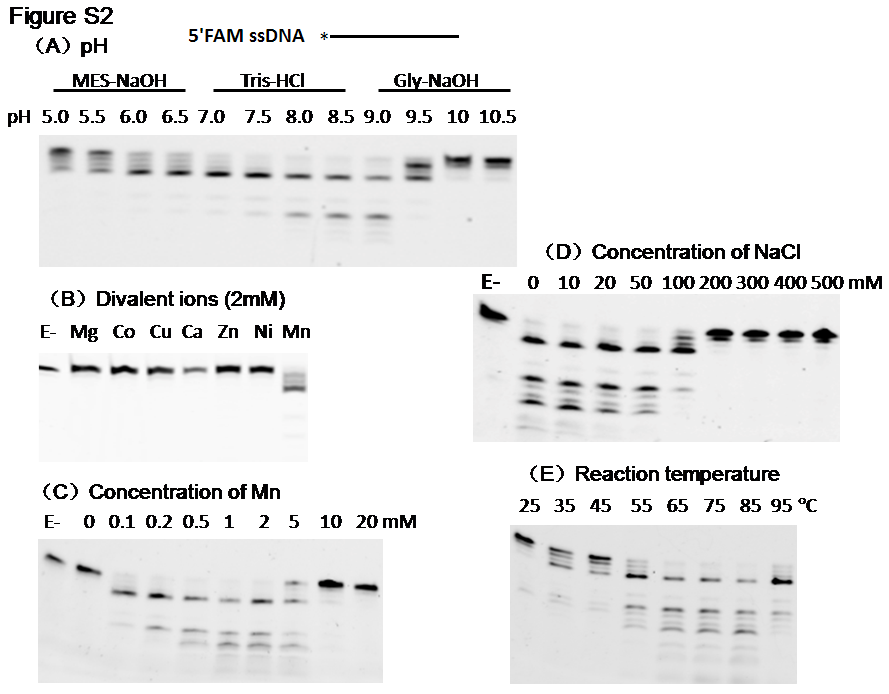
**

**Figure S2. Biochemical characterization of MjaRecJ2.** The same optimized parameters shown in Figure S1 were considered. During biochemical characterization of 3’🡪5’ exonuclease activity, the ssDNA substrate (100 nM) was incubated with 50 nM MjaRecJ2 at 55 °C (except for the assay of reaction temperature) for 20 min. The pH of the reaction buffer was optimized in basic buffer consisting of 20 mM NaCl, 1 mM DTT, and 2.0 mM MnCl_2_. After pH optimization, effects of divalent ions (2.0 mM) on activity were characterized in a buffer with pH 8.5, and optimal concentration of Mn^2+^ was further assayed within the range of 0.1–20 mM. Ion strength was optimized in the buffer consisting of 20 mM Tris-HCl, 1.0 mM Mn^2+^, 1 mM DTT, and increasing concentrations of NaCl. Finally, reaction temperature was characterized in an optimized buffer consisting of 20 mM Tris-HCl (pH 8.5), 20 mM NaCl, 1.0 mM Mn^2+^, and 1 mM DTT. The products in each image were quantified to construct the curve shown in Figure 2.


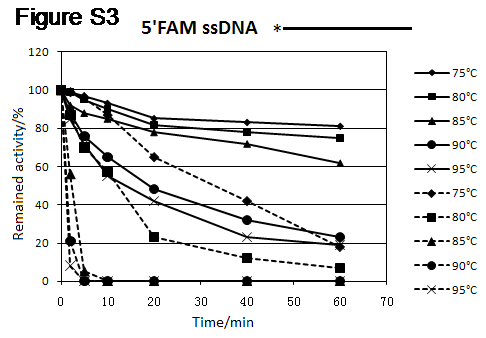


**Figure S3 Thermostabilities of MjaRecJs.** Thermostabilities of the two RecJ nucleases were characterized in their respective optimized reaction buffer. Reaction mixtures were preheated at different temperatures with increasing time and without substrate addition. Reactions were performed at 55 °C for 20 min after adding 23 nt ssDNA substrate. The optimized buffer of MjaRecJ2 was consisted of 20 mM Tris-HCl (pH 8.5), 20 mM NaCl, 1.0 mM Mn^2+^, and 1 mM DTT. The optimized MjaRecJ1 buffer was consisted of 20 mM Tris-HCl (pH 8.0), 50 mM NaCl, 2.0 mM Mn^2+^, and 1 mM DTT. The remaining activities were calculated as percentage of no-heat enzymes whose activities are considered as 100%. The dashed lines represent RecJ1, and solid lines represent MjaRecJ2.


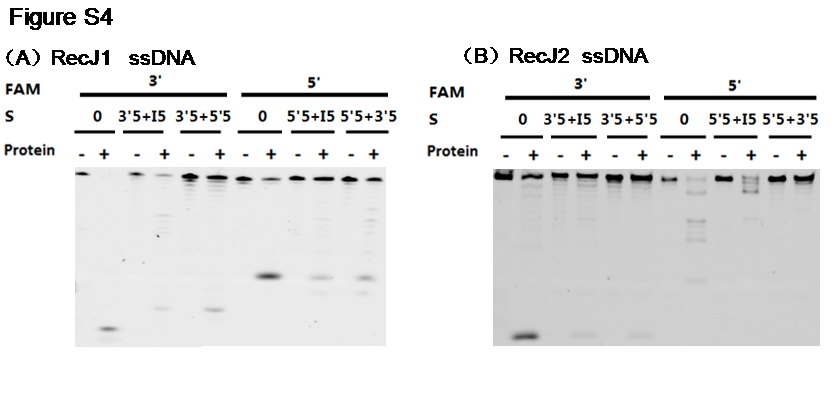


**Figure S4. Hydrolysis polarity of two MjaRecJs confirmed by special phosphothioate-modified substrates.** Two MjaRecJs (40 nM MjaRecJ1 or 50 nM MjaRecJ2) were incubated with 100 nM 23 nt ssDNA substrates with different phosphothiate modifications at 55 °C for 20 min in their respective reaction buffer. The ssDNA substrates underwent successive phosphothioate modifications in the middle and/or termini of phosphate (deoxy)ribose backbones. The values 3’5 and 5’5 indicate five successive phosphothioate groups on the 5’ or 3’ end, respectively. I5 indicates five successive phosphothioate groups in the middle of ssDNA.


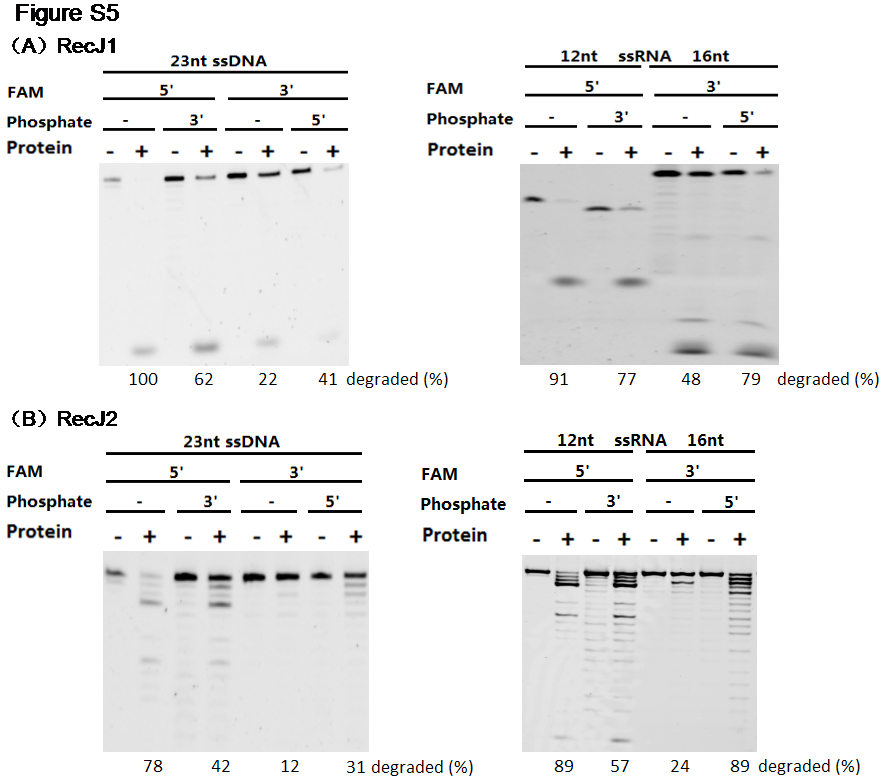


**Figure S5. Effect of terminal phosphate group on *M. jannaschii* RecJs activity.** Two MjaRecJs (40 nM MjaRecJ1 or 50 nM MjaRecJ2) were incubated with 100 nM 23 nt ssDNA or 12 or 16 nt ssRNA substrates at 55 °C for 20 min in their respective reaction buffer. The substrates contained a 3’or 5’ terminal phosphate group. The degraded amount of substrate was quantified and listed at the bottom of the panel.


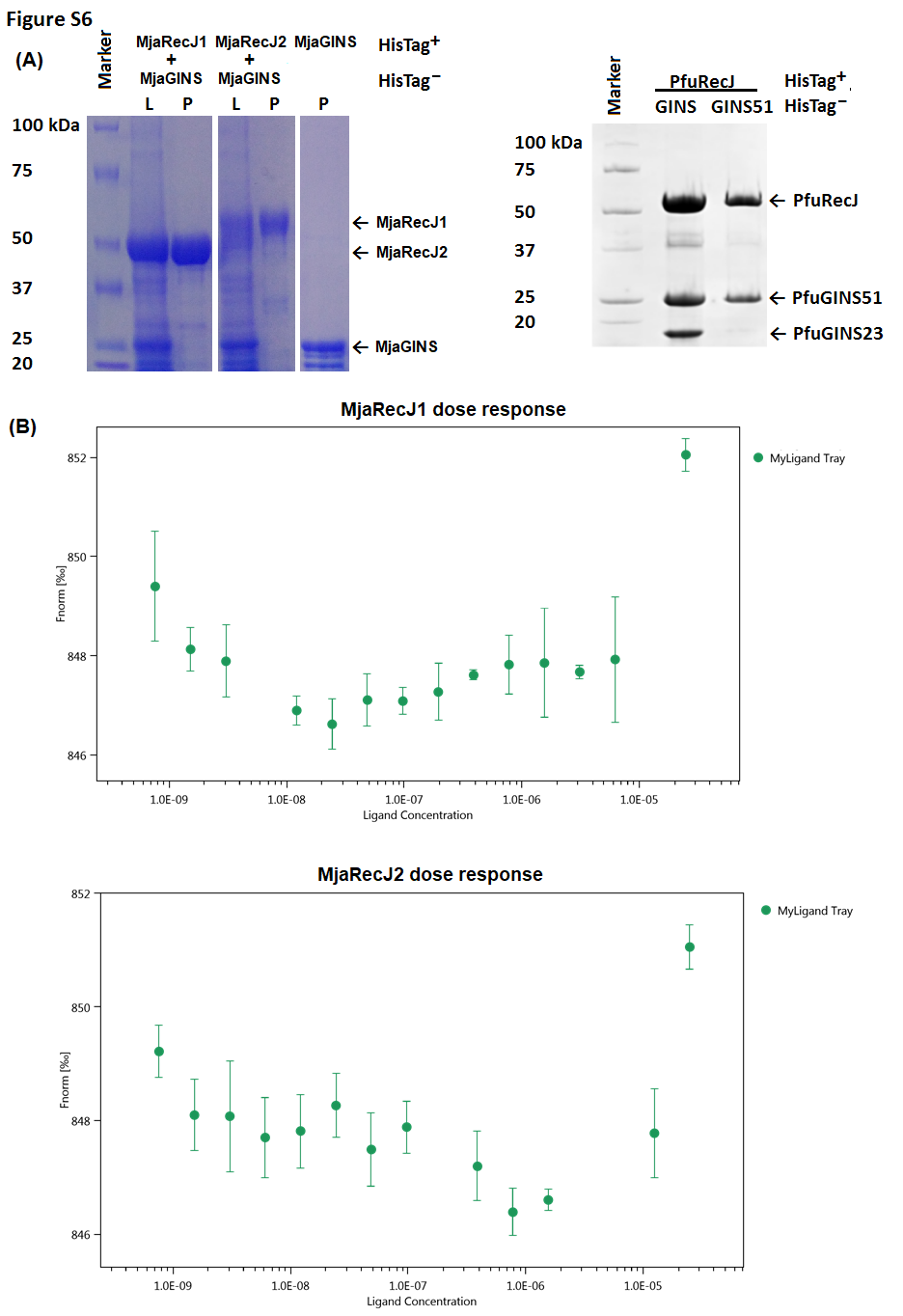


**Figure S6. Interaction identification of MjaRecJs and MjaGINS. (A) MjaGINS could not pulldown MjaRecJ1 or MjaRecJ2.** Pulldown of MjaGINS by HisTag MjaRecJs were performed using the induced *E. coli* cells that express both MjaRecJ and MjaGINS via the pCDFDuet vector. The letters L and P, on the top of gels of pulldown experiments, indicate cell lysates and purified proteins, respectively. A positive control experiment, pulldown of PfuGINS subcomplex via HisTag PfuRecJ, was performed in the presence of excess GINS or GINS51. (B) Microscale Thermophoresis (MST) assays of MjaGINS and MjaRecJ1 (top panel) and MjaRecJ2 (bottom panel). MST measurements were performed on a Monolith NT.115 instrument (NanoTemper Technologies). The MjaGINS was dissolved in a buffer solution containing 50 mM HEPES, pH 8.0 and labeled with fluorescent group, and increasing concentrations of MjaRecJ1 and MjaRecJ2 were used as the ligands. The nonlinear fitting curves could not performed for MST data, so that it is impossible to calculate the values of K_d_, indicating that clear interaction does not exist between MjaGINS and MjaRecJ1 and MjaRecJ2, respectively. In other words, our MST results also confirmed that MjaGINS does not interact with two MjaRecJs.


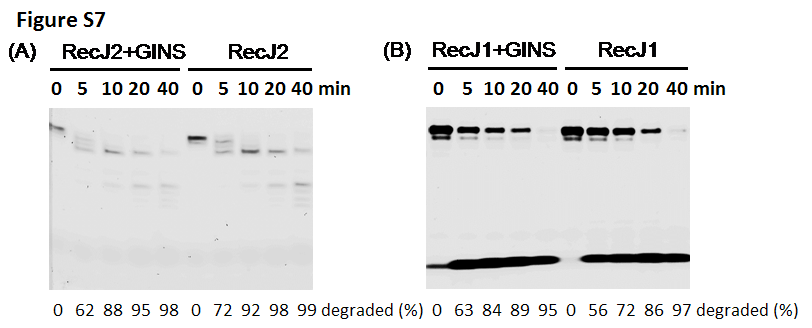


**Figure S7 Effect of MjaGINS on MjaRecJs activity.** Two MjaRecJs (40 nM MjaRecJ1 or 50 nM MjaRecJ2) were incubated with 100nM 23 nt ssDNA substrate at 55 °C for 0, 5, 10, 20, and 40 min in their respective reaction buffer and in the presence or absence of 200 nM MjaGINS. The degraded amount of substrate was quantified at each time and listed at the bottom of the panel.

**Supplementary Table**

**Table S1.** Oligonucleotides used in this research.

| **Names** | **Sequences (5’-3’)** | **Comments** |
| --- | --- | --- |
| DD286 | *TCCGATAGCCAGATATCTTGACA | Figure 1–3, S1-S3,S6 |
| JL510 | *uccgauagccagauaucuugacu |  |
| DD305 | *TCCGATAGCCAGATATCTTGACA | Figure 4 |
| DD306 | TCCGATAGCCAGATATCTTGACA* |  |
| DD264 | *csasgscscsasgsgsusgsuscsuscsascsu |  |
| DD265 | asgscscsgsascsuscsgscscsascsasgsu* |  |
| DD295 | TCCGATAGCCAGATATCTTGACA* | Figure S4 |
| DD303 | TCCGATAGCsCsAsGsAsTATCTsTsGsAsCsA* |  |
| DD304 | TsCsCsGsAsTAGCCAGATATCTsTsGsAsCsA* |  |
| DD286 | *TCCGATAGCCAGATATCTTGACA |  |
| DD300 | *TsCsCsGsAsTAGCCsAsGsAsTsATCTTGACA |  |
| DD301 | *TsCsCsGsAsTAGCCAGATATCTsTsGsAsCsA |  |
| DD295 | TCCGATAGCCAGATATCTTGACA* | Figures 5 and S5 |
| DD298 | pTCCGATAGCCAGATATCTTGACA* |  |
| DD286 | *TCCGATAGCCAGATATCTTGACA |  |
| DD297 | *TCCGATAGCCAGATATCTTGACAp |  |
| DD254 | cgagcggagaugacgg* |  |
| DD257 | pcgagcggagaugacgg* |  |
| DD247 | *cggagaugacgg |  |
| DD256 | *cggagaugacggp |  |
| DD282 | *CGAT | Figure 6 |
| DD283 | *TCCGAT |  |
| DD284 | *TCCGATAGCCAG |  |
| DD285 | *TCCGATAGCCAGATATC |  |
| DD286 | *TCCGATAGCCAGATATCTTGACA |  |
| DD287 | *TCCGATAGCCAGATATCTTGTGAGCGTGGG |  |
| DD288 | *AGGCTGCGGTCGAGTTGACAGCACTGCACGCATTACTGAGCT |  |
| DD289 | *CTCCAGTGGTGTTCGGCTCCGATAGCCAGATATCTTGTGACGTGACGTGCGTAATGAC |  |
| DD245 | *acgu |  |
| DD246 | *ugacgu |  |
| DD247 | *cggagaugacgg |  |
| DD248 | *cgagcggagaugacgg |  |
| DD288 | *AGGCTGCGGTCGAGTTGACAGCACTGCACGCATTACTGAGCT | Figure 7 |
| JL389 | AGCTCAGTAATGCGTGCAGTGCTGTCAA |  |
| JL388 | AGCTCAGTAATGCGTGCAGTGCTGTCAACTCGACCGCAGCCTTTTTT |  |
| JL387 | AGCTCAGTAATGCGTGCAGTGCTGTCAAGAGCTGGCGTCGGA |  |
| JL386 | AGCTCAGTAATGCGTGCAGTGCTGTCAACTCGACCGCAGCCT |  |
| JL390 | TCGAGTCATTACGCTGCAGTGCTGTCAACTCGACCGCAGCCT |  |
| JL391 | TGCAGTGCTGTCAACTCGACCGCAGCCT |  |
| JL392 | TTTTTAGCTCAGTAATGCGTGCAGTGCTGTCAACTCGACCGCAGCCT |  |
| MJ0831F | GGCTCCCATATGATGGAAAAACTTAAAGA | Insertion of Mj0831 into pDEST17 |
| MJ0831R | GGATCCACTAGTTTATCTCCTTAACTGCTC |  |
| MJ0977F | GGCTCCCATATGGAAAATTGGGTAGAATT | Insertion of Mj0977 into pDEST17 |
| MJ0977R | GGATCCACTAGT TTATCTCAAAGCAATCTCAA |  |
| MJ0831-DHH-F | GCCGCTGCTCCTCCAGTTATAAAAGATA | Changing residues DHH of Mj0977 into AAA |
| MJ0831-DHH-R | AGGAGCAGCGGCTAATATAATTGCATTAAAG |  |
| MJ0977-DHH-F | GCCGCTGCCCAACCAGAGGAGATAAAG | Changing residues DHH of Mj0977 into AAA |
| MJ0977-DHH-R | TTGGGCAGCGGCTAAGATAATAATTTTGTCT |  |

^a^ Asterisks denote the fluorescein (6-FAM) moiety at the 5’ or 3’ end. Lowercase letters represent RNA, and uppercase letters represent DNA. The phosphorothioate modifications are denoted by letter s, and the phosphate groups are denoted by letter p. ^b^ Amino acid substitutions were introduced into MjaRecJs, as described previously [14].
